# Value of CEUS features in diagnosing thyroid nodules with halo sign on B-mode ultrasound

**DOI:** 10.1186/s12880-023-00966-y

**Published:** 2023-01-21

**Authors:** Xue-jun Chen, Lin-jin Huang, Feng Mao, Hai-xia Yuan, Xi Wang, Qing Lu, Cai-hong Dong

**Affiliations:** 1 Department of Ultrasound, Zhongshan Hospital (Xiamen), Fudan University, Xiamen, 361015 Fujian Province China; 2 Department of Ultrasound, Zhongshan Hospital, Fudan University, Shanghai, 200030 China

**Keywords:** Contrast-enhanced ultrasound, Thyroid nodule, Halo

## Abstract

**Background:**

The results of halo sign in the differential diagnosis of thyroid nodules were conflicting, and the value of contrast-enhanced ultrasound (CEUS) in characterization of thyroid nodules with halo has not been fully evaluated. This study was therefore designed to investigate the value of contrast-enhanced ultrasound features in the differential diagnosis of thyroid nodules with halo sign on B-mode ultrasound.

**Material and methods:**

Seventy-four consecutive thyroid nodules with halo sign on B-mode ultrasound were pathologically confirmed by surgery or fine needle aspiration, including 43 benign and 31 malignant lesions. All these lesions underwent pre-operative CEUS examination. The CEUS features, including enhanced time, enhanced intensity and homogeneity, and presence of enhancing ring, were compared between benign and malignant ones.

**Results:**

Enhanced intensity was significant different between benign and malignant lesions with halo. Hypo-enhancement was more frequently detected in malignant nodules than that in benign ones, compared with iso-enhancement and hyper-enhancement (*p* = 0.013, and = 0.014, respectively). Detection rate of high-enhancing ring was significantly higher in benign nodules than that in malignant group (*p* = 0.001). While in nodules > 10 mm, only high-enhancing ring was the distinguishing feature between benign and malignant nodules.

**Conclusions:**

Enhanced intensity and high-enhancing ring may be helpful in the differential diagnosis of thyroid nodules with halo sign on B-mode ultrasound.

## Introduction

The detection of thyroid carcinoma is increasing rapidly due to advanced ultrasound (US) modalities and techniques. Conventional US was considered as the first line screen tool, however, there were considerable overlaps of US appearance between benign and malignant thyroid nodules, and single US feature often showed inadequate sensitivity and specificity for the differential diagnosis. In order to improve diagnostic performance, international and domestic professional committees presented several thyroid imaging reporting and data systems (TI-RADS) for clinical risk stratification of thyroid nodules. However, none of these guidelines cover all features. Halo sign was a common US finding defined as a hypo-echoic rim surrounding the solid thyroid masses [1], which used to be recognized as a predictor of benign nodules. However, during clinical practice, it has also been reported in malignant ones [2]. Therefore, effective complementary modalities are required for the differentiation between benign and malignant thyroid nodules with halo sign.

Contrast enhanced ultrasound (CEUS) can provide nodules microvascular perfusion information due to its “pure blood pool” nature. Most previous studies indicated that CEUS features could facilitate high predictive value of malignant thyroid nodules, such as heterogeneous and hypo-enhancement [3, 4]. Recently, some studies reported that peripheral enhancing ring on CEUS was an important characteristic feature for differential diagnosis, despite of conflict results. Zhang et al. demonstrated that ring enhancement was predictive of benign nodules [5], while another retrospective study reported that peripheral irregular ring enhancement was helpful to detect malignancy [6]. Notably, the definition of peripheral ring enhancement was not clearly defined in the previous literature. Therefore, in present study, we include in the definition of ring enhancement of both high-enhancing ring and low-enhancing rings.

So far, the value of CEUS in characterization of thyroid nodules with halo has not been fully evaluated; furthermore, no prior literatures have ever investigated the correlation between CEUS ring enhancement and B-mode halo. Therefore, the aim of this retrospective study was to determine whether there are significant differences in enhanced patterns between benign and malignant lesions with halo sign, and to explore the value of CEUS features in the differential diagnosis of these nodules.

## Materials and methods

### Patients

**Fig. 1:**
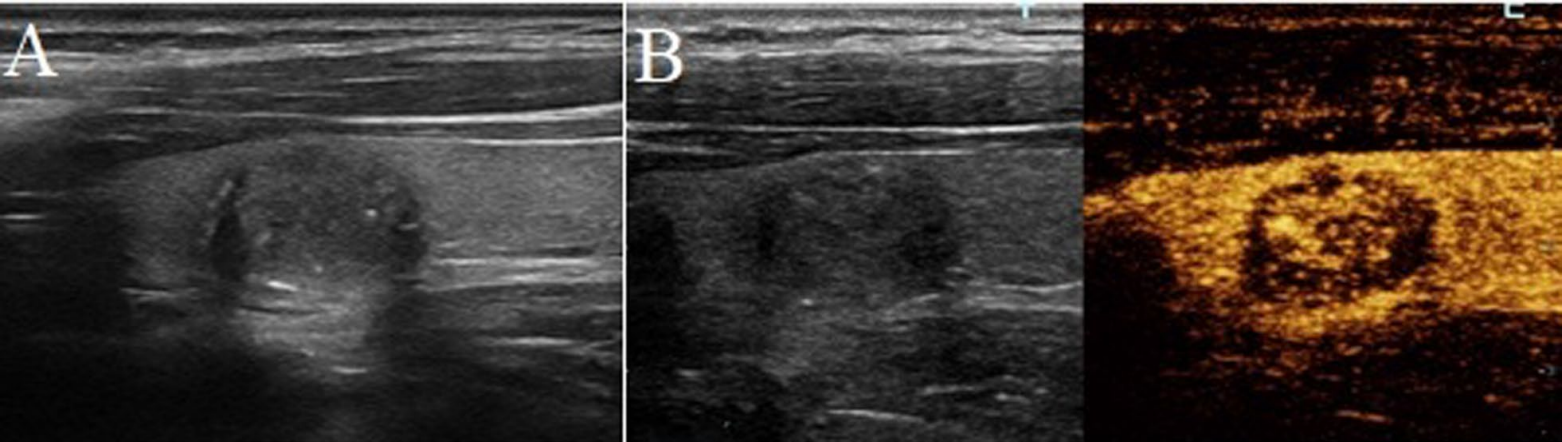
A papillary thyroid carcinoma in a 40-year-old woman. **A** Greyscale ultrasound showed that there was a halo surrounding the thyroid nodule. **B** Contrast-enhanced ultrasound revealed hypo-enhancement at peak, with a irregular low-enhancing ring (at the 20th second after the injection of contrast agent)

**Fig. 2:**
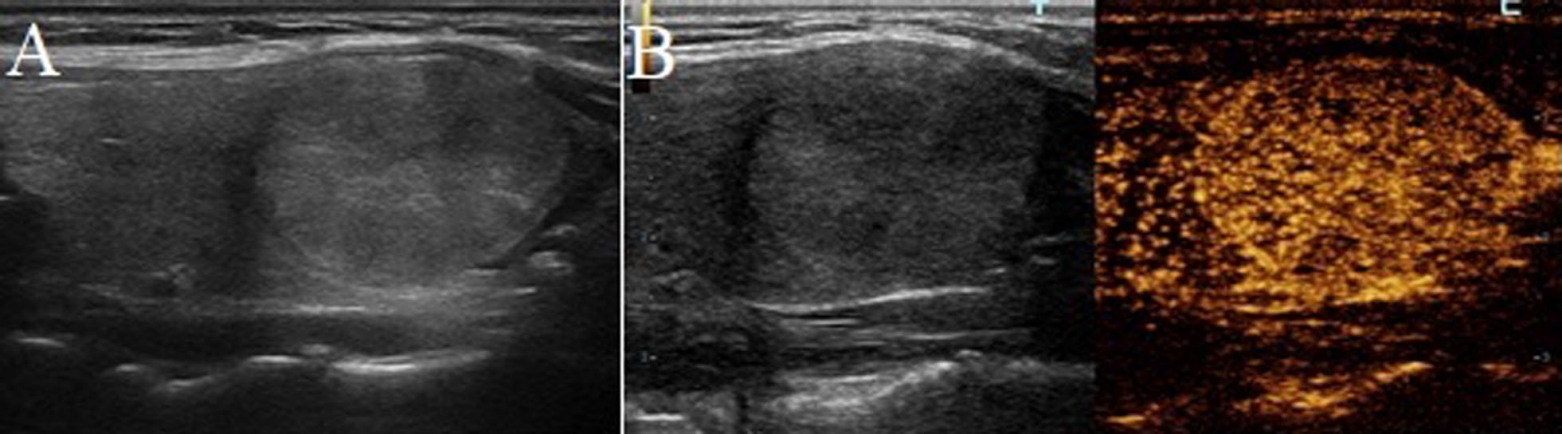
A follicular adenoma in a 53-year-old woman. **A** Greyscale ultrasound showed that there was a halo surrounding the thyroid nodule. **B** Contrast-enhanced ultrasound revealed regular high-enhancing ring (at the 24th second after the injection of contrast)

This retrospective study was approved by the Research Ethics Committee and Review Board of our institute, and written informed consent was obtained from all participants. From August 2019 to June 2020, a total of 750 patients with 825 thyroid nodules underwent conventional US and CEUS examination in our institute. The inclusion criteria were as follows: (a) adults older than 18 years; (b) nodules with halo sign (complete hypo-echoic rim around nodule, either regular or irregular) on B-mode US (Figs. 1A, 2A); (c) pathologically confirmed by fine needle aspiration (FNA) or post-operative histopathologic examination.

The exclusion criteria were as follows: (a) large nodules without peri-nodular normal parenchyma as a reference on US plane; (b) nodules without definite diagnosis (Bethesda I or III on FNA); (c) benign nodules without repeated FNA when nodules with Bethesda category II or with an enlargement of the nodule size and a change in American College of Radiology (ACR) TI-RADS category until the most recent clinical follow-up; (d) video recording with no black screen before contrast agent arrival; (e) technical problems (e.g., wiggly recording, or fragile swallow). Totally, 74 patients (23males and 51 females) with 74 nodules were included in this study, with median age 42 years (IQR 35–65 years) and median diameter 10 mm (IQR 8-15 mm).

### Conventional US and contrast-enhanced examinations

All US examinations were performed by two radiologists with more than 5 experience years in CEUS examination, independently, using machinesa Resona R7S system (Mindray Medical International, Shenzhen, China) equipped with a L14-5WU transducer for conventional US and a L11-3U transducer for CEUS.

On conventional US, once the target nodule was determined, the following characteristics were recorded, including the size (maximum diameter), location, internal structures, echogenicity, margin, shape, presence or absence of microcalcification and presence or absence of halo sign.

On CEUS examination, the largest plane of the target nodule was selected, and then the transducer was switched to the harmonic CEUS mode. Low mechanical index (MI < 0.10) was set to minimize microbubble destruction and artificial signal loss. A single focus was always set under the target nodule. A dose of 2.0 mL sulfur hexafluoride microbubble contrast agent SonoVue (Bracco S.p.A Inc., Milan, Italy) was then injected via antecubital vein in bolus, followed by additional 5.0 mL 0.9% sodium chloride solution as a flush. The timer on the US device was started simultaneously with the injection of contrast agent, and CEUS process was recorded continuously for at least 120s and digitally stored as raw data.

### Imaging interpretation

Another two experienced radiologists with more than 15-years-experience in thyroid US interpretation reviewed the conventional US and CEUS examinations in consensus to identify image features. Both of them were blinded to the interpretation of the previous radiologists. The CEUS features of thyroid nodules were classified as follows: (1) wash-in and wash-out times were classified as early, simultaneous and late; (2) At peak enhancement, enhanced intensity was classified as hyper-, iso-, or hypo-enhancement compared with the surrounding parenchyma; (3) According to the enhanced homogeneity at peak, homogeneous enhancement was defined as an occupation with a full enhancement with same intensity, while heterogeneous enhancement was defined as an enhanced lesion with inconsistent enhanced intensity; (4) The presence of enhanced defect was defined as the presence of unenhanced areas within the nodule; (5) As for the enhanced pattern, centripetal enhancement was defined as the contrast agent entered from the periphery of the lesion to the center, eccentrical enhancement was defined as nodule enhanced from the center of the lesion to the periphery, and comprehensive enhancement was defined that both the central and peripheral areas of the nodule enhanced synchronously; (6) The presence of peripheral enhancing rings were divided into (a) low-enhancing ring (hypo-enhanced rim compared with the intensity of the nodule) (Fig. 1B); (b) high-enhancing ring (hyper-enhanced rim compared with the intensity of the nodule) (Fig. 2B); (c) absence of peripheral ring.

### Reference standard

The interval between US/CEUS examination and surgery or FNA did not exceeded 4 weeks, during which no clinical intervention was performed. The reference standard was as follows: (1) all malignant nodules were pathologically confirmed by surgery; (2) For benign nodules, the diagnosis was confirmed by surgery, or FNA repeated 2 times with benign results (Bethesda category II), or a benign result on FNA and no enlargement of the nodule size and no change in ACR TI-RADS category until the most recent clinical follow-up, at least 1 year’s follow-up.

### Statistical analysis

The statistical analysis was performed with SPSS version 26.0 software (IBM Corporation, Armonk, NY). Continuous data was reported as median with interquartile range (IQR). Categorical data was shown as frequency and percentages. The chi-squared test (χ^2^) was used to compare categorical data. Values of *p* < 0.05 (two tailed) were considered statistically significant. Pairwise comparisons were conducted for each category of the categorical data with three categories. Bonferroni correction was used for comparison of the three categories, and values of *p* < 0.017 (0.05/3) were considered statistically significant. For the significant features between benign and malignant nodules, the sensitivity and specificity were calculated. Numerous previous studies found that the enhancement patterns of thyroid nodules were affected by nodule size [7, 8]. Thus, nodules in our study were divided into two subgroups (≤ 10 mm and > 10 mm) according to the maximum lesion diameter for subgroup analysis.

## Results

### Pathological results

Pathology examination demonstrated that 31 (41.9%) nodules were malignant, including 30 papillary thyroid carcinomas and 1 medullary carcinoma, and 43 (58.1%) nodules were benign. Among the 43 benign nodules, 15 were surgically proved (5 nodular goiters, 10 follicular adenomas) and 28 were confirmed by FNA and follow-up.

### Comparisons of CEUS characteristics between benign and malignant thyroid nodules

Comparisons of CEUS characteristics between benign and malignant thyroid nodules with halo sign are shown in Table 1. Peak enhanced intensity was significantly different between benign and malignant thyroid nodules (*p* = 0.016). Hypo-enhancement was more common in malignant nodules than that in benign ones compared with iso-enhancement and hyper-enhancement (*p* = 0.013 and = 0.014, respectively). Patterns of peripheral enhancing rings were significantly different between benign and malignant nodules (*p* = 0.001). High-enhancing ring was significantly more common in benign lesions than that in malignant ones (*p* = 0.001). No significant difference was detected in wash-in time, enhancement homogeneity, enhancement defect, enhancement pattern, and wash-out time between benign and malignant nodules (All *p* > 0.05).

**Table 1:** CEUS characteristics of benign and malignant thyroid nodules

Indicator	Pathology		*p*
	Benign	Malignant	
Wash-in			0.216*
Earlier	7 (16.3%)	1 (3.2%)	
Meantime	30 (69.8%)	24 (77.4%)	
Later	6 (14.0%)	6 (19.4%)	
Intensity			0.016*
Hypo-enhancement	2 (4.7%)	9 (29.0%)	0.013^#^
Iso-enhancement	27 (62.8%)	14 (45.2%)	0.86^#^
Hyper-enhancement	14 (32.6%)	8 (25.8%)	0.014^#^
Homogeneity			0.212*
Homogeneity	40 (93.0%)	25 (80.6%)	
Heterogeneity	3 (7.0%)	6 (19.4%)	
Enhanced defect			0.052*
Without	29 (67.4%)	27 (87.1%)	
With	14 (32.6%)	4 (12.9%)	
Enhanced pattern			0.699*
Entire	40 (93.0%)	27 (87.1%)	
Centripetal	1 (2.3%)	2 (6.5%)	
Eccentrical	2 (4.7%)	2 (6.5%)	
Enhancing ring			0.001*
Without	18 (41.9%)	14 (45.2%)	0.008^#^
High	11 (25.6%)	0 (0.0%)	0.001^#^
Low	14 (32.6%)	17 (54.8%)	0.454^#^
Wash out			0.801*
Earlier	10 (23.3%)	8 (25.8%)	
Later	33 (76.7%)	23 (74.2%)	

*Determined with the χ^2^ test

^#^Bonferroni correction was used for comparisons of the three categories, and values of *p* < 0.017 (0.05/3) were considered statistically significant for the pairwise comparisons

### Comparisons of CEUS characteristics in thyroid nodules with different size

Comparisons of CEUS characteristics in thyroid nodules with different size are shown in Table 2. Patterns of peripheral enhancing rings were significantly different between benign and malignant thyroid nodules in the group of nodules > 10 mm (*p* = 0.004), while there was no significant difference of enhanced features in group of nodules ≤ 10 mm (all *p* > 0.05). In group of nodules > 10 mm, high-enhancing ring was more common in benign nodules than that in malignant nodules (*p* = 0.003).

**Table 2:** Comparison of CEUS characteristics in nodules with different tumor size

Indicator	≤ 10 mm		*p*	> 10 mm		*p*
	Benign	Malignant		Benign	Malignant	
Wash-in			0.736*			0.161*
Earlier	0 (0.0%)	0 (0.0%)		7 (31.8%)	1 (11.1%)	
Meantime	15 (71.4%)	17 (77.3%)		15 (68.2%)	7 (77.8%)	
Later	6 (28.6%)	5 (22.7%)		0 (0.0%)	1 (11.1%)	
Intensity			0.124*			0.365*
Hypo-enhancement	2 (9.5%)	8 (36.4%)		0 (0.0%)	1 (11.1%)	
Iso-enhancement	14 (66.7%)	10 (45.5%)		13 (59.1%)	4 (44.4%)	
Hyper-enhancement	5 (23.8%)	4 (18.2%)		9 (40.9%)	4 (44.4%)	
Homogeneity			1.000*			0.063*
Homogeneity	19 (90.5%)	19 (86.4%)		21 (95.5%)	6 (66.7%)	
Heterogeneity	2 (9.5%)	3 (13.6%)		1 (4.5%)	3 (33.3%)	
Enhanced defect			0.185*			0.826*
Without	17 (81.0%)	21 (95.5%)		12 (54.5%)	6 (66.7%)	
With	4 (19.0%)	1 (4.5%)		10 (45.5%)	3 (33.3%)	
Enhanced pattern			1.000*			0.290*
Entire	18 (85.7%)	19 (86.4%)		22 (100.0%)	8 (88.9%)	
Centripetal	1 (4.8%)	2 (9.1%)		0 (0.0%)	0 (0.0%)	
Eccentrical	2 (9.5%)	1 (4.5%)		0 (0.0%)	1 (11.1%)	
Enhancing ring			0.763*			0.004*
Without	9 (42.9%)	11 (50.0%)		9 (40.9%)	3 (33.3%)	0.221^#^
High	1 (4.8%)	0 (0.0%)		10 (15.5%)	0 (0.0%)	0.003^#^
Low	11 (52.4%)	11 (50.0%)		3 (13.6%)	6 (66.7%)	0.087^#^
Wash out			0.656*			0.287*
Earlier	6 (28.6%)	4 (18.2%)		4 (18.2%)	4 (44.4%)	
Later	15 (71.4%)	18 (81.8%)		18 (81.8%)	5 (55.6%)	

*Determined with the χ^2^ test

^#^Bonferroni correction was used for comparisons of the three categories, and values of *p* < 0.017 (0.05/3) were considered statistically significant for the pairwise comparisons

### Diagnostic performance of CEUS characteristics in thyroid nodules with halo sign

With hypo-enhancement as the criteria for the diagnosis of malignant nodules, the specificity was high (95.35%) and the sensitivity was poor (29.03%). With high-enhancing ring as the criteria for the diagnosis of benign nodules, the sensitivity and specificity were 25.58%, and 100.0%, respectively.

## Discussion

In present study, we compared CEUS features between malignant and benign thyroid nodules with halo sign on conditional US. Enhanced intensity and ring enhancement were found significantly different between benign and malignant nodules. The value of CEUS features in the differential diagnosis of thyroid nodules with halo sign was firstly discussed.

Timely diagnosis of thyroid nodules is of considerable importance for clinical treatment and may effectively improve the prognosis. Conventional US is usually considered as a valuable diagnostic method for thyroid nodules. However, there are considerable overlaps of US features between benign and malignant ones, for instance, chronic lymphocytic thyroiditis may present as hypoechoic with an instinct margin and microcalcifications, and carcinomas may also present halo sign. Therefore, conventional US has limitations for such diagnostic dilemma. FNA is regarded as the golden standard before surgery, but this invasive diagnostic method has false negative outcomes and up to 30% indeterminate cytology results [9, 10].The American Thyroid Association (ATA) and ACR have presented the risk stratification systems of thyroid nodules and provide guidance for FNA [11, 12], however, with relatively low specificity and high heterogeneity among sonographers [9]. CEUS can facilitate better diagnostic performance for thyroid nodules and reducing some unnecessary over-diagnosis, and has been introduced to clinical practice as a complementary modality for conditional US in the differential diagnosis of thyroid nodules [4, 13].

Halo sign was inferred to the peri-nodular hypo-echoic rim, which was due to the compressed surrounding capsular tissue or inflammatory infiltration or both on pathological examination and has been reported in various pathological types of thyroid nodules, either benign or malignant [14]. The results of published literatures regarding to the halo sign in the differential diagnosis of thyroid nodules were conflicting, with some studies demonstrating halo sign as the predictor of benign nodules [15], while no such association in other studies [16]. This may be due to the indistinct definition of halo sign among different studies, in which the intactness and uniform of halo were not mentioned. In present study, we explicitly defined halo sign as a complete hypo-echoic ring around the thyroid nodule, either regular or irregular. Of the 41.9% (31/74) nodules with complete halo were confirmed to be carcinomas. Therefore, halo sign is not a specific characteristic for benign thyroid nodules and it is crucial for new techniques to accurate differential diagnosis of these thyroid nodules.

Though numerous studies have proved that CEUS features demonstrated good sensitivity and specificity in diagnosing thyroid cancer, there are still no widely accepted diagnostic guidelines, resulting in its inability of wide clinical application. Most prior studies focused on enhanced modes and intensity, indicating heterogeneous or hypo-enhancement as predictors of malignancy. Our study acquired similar results that the enhanced intensity was significantly different between benign and malignant thyroid nodules. With hypo-enhancement as the criteria for the diagnosis of malignant nodules, the specificity was high (95.35%), however, the sensitivity was poor (29.03%), due to the relatively high proportion (45.2%) of iso-enhancement detected in malignant nodules. The high frequency of iso-enhancement seemed to be not exactly the same as most previous studies, in which iso-enhancement on CEUS usually suggested benignity [17]. We speculated that the result might be related to the difference of inclusion criteria. In present study, nodules without halo were excluded, and it is possible to infer that surrounding capsular tissue or inflammatory infiltration which resulted in halo sign may affect the original uneven proliferation of tumor cell and immaturation of tumor vessel inside the nodules. The present study showed that the enhanced time, pattern, homogeneity, enhanced defect and washout time did not differ between benign and malignant thyroid nodules, which was not consistent with previous reports. This may be due to the smaller size of nodules in our study, in which nearly half of the nodules (43/74) measuring ≤ 10 mm in maximum diameter. Quite a few patients with nodule size ≤ 10 mm were subjected to FNA or surgery, which is a common phenomenon in many cities in China, related to the patients’ anxiety and doctors’ worry about missed diagnosis. Foschini et al. reported CEUS enhanced features differed among nodules with different size. Enhanced heterogeneity reflected the uneven distribution of tumor blood vessels in malignant nodules and it would be more obvious with the nodule enlargement [18]. Our results can also be supported by another study that demonstrated CEUS had no significant advantage in characterizing PTMC [19].

Recently, several studies reported that peripheral enhancing rings may provide valuable diagnostic information, which could further improve the diagnostic accuracy [5, 20], which was also approved in present study. Peripheral enhanced ring is speculated to be associated with capsular and peripheral compressed parenchymal vessels around the nodule, which seem to be similar with the mechanism of halo sign on conventional US. In most previous studies only high-enhancing ring was defined as peripheral enhanced pattern. However, in present study, enhancing rings were distinctly divided into high-enhancing ring and low-enhancing ring. We identified that high-enhancing rings were more frequent in benign nodules, which was in concordance with one retrospective study that implied that peripheral regular high-enhancing rings were almost found in benign thyroid nodules, especially in adenoma and nodular goiter [6]. Adenomas and nodular goiter are frequently accompanied with cystic areas, which could reduce the compression force, the capsular vessels or the compressed tissues around lesion might result in the high-enhancing rings. In our study, none of the 11 lesions with high-enhancing ring turned out to be malignant, thus we inferred that high-enhancing ring would be a predictor of benignity. In present study, with high-enhancing ring as the criteria for the diagnosis of benign nodules, the specificity reached 100.0%, despite of the low sensitivity (25.58%). This result was meaningful, since it can reduce the false positive diagnosis and avoid unnecessary FNA for these nodules. However, due to the relatively small sample size, the peripheral enhanced patterns were not divided into more categories, such as regular and irregular high-enhancing rings and regular and irregular low-enhancing rings, which would be further studied with larger study population in the future.

Taking the nodule size into account, we further evaluated enhanced features between benign and malignant nodules according to different size, and found that high-enhancing ring was more frequently detected in benign nodules than that in malignant one in group > 10 mm. Alternatively, such difference was not significant between benign and malignant ones ≤ 10 mm. The result may be supported by numerous published reports, which pointed out CEUS features considerably overlapped in benign and malignant nodules measuring ≤ 10 mm. Li et al. reported that CEUS had no significant advantage in differentiating PTMCs as they usually present atypical enhancement features [16]. The reasons may be hypothesized that when the tumors are small, differences in the microvessel density may not be evident between benign and malignant lesions. Furthermore, overlapping appearance may also be associated with the instrument sensitivity, the parameters and adjustments, especially when nodules are small. In present study, though the frequency of low- enhancing ring in malignant nodules was higher than that in benign ones in the group > 10 mm (66.7% vs. 13.6%), the difference was not significant. This might be due to the small sample size in nodules > 10 mm (22 benignities and 9 malignancies), and the value of low-enhancing ring should be further analyzed.

However, this study truly has some limitations. First, a selection bias might be present, because only nodules with halo sign were included. Second, as the present study was with small sample size, in which some frequency < 5, additional multi-center studies and larger sample sizes are needed in the future. Third, the malignant thyroid nodules were mainly papillary thyroid carcinoma, while only one is medullary carcinoma. Other histological types are needed to be included and analyzed in the future. Finally, the present study is a retrospective analysis, which had inherent basis. Thus a prospective well-designed study is needed for more accurate assessment of the relation between the CEUS features and thyroid nodules with halo sign.

## Conclusion

In conclusion, in thyroid nodules with peripheral halo, enhanced intensity combined with peripheral CEUS patterns can provide useful information for differential diagnosis of benign and malignant ones, which has certain diagnostic value.

## Data Availability

The datasets used and/or analyzed during the current study available from the corresponding author on reasonable request.

## Abbreviations

US: Ultrasound
CEUS: Contrast-enhanced ultrasound
TI-RADS: Thyroid imaging reporting and data system
FNA: Fine needle aspiration
ATA: American Thyroid Association
ACR: American College of Radiology
